# Analysis of Chemical Composition, Amino Acid Content, and Rumen Degradation Characteristics of Six Organic Feeds

**DOI:** 10.3390/ani12060682

**Published:** 2022-03-09

**Authors:** Chenglong Luo, Donghai Wang, Na Lu, Haiqing Li, Gaofei Liu, Zhijun Cao, Hongjian Yang, Shengli Li, Xiong Yu, Wei Shao, Wei Wang

**Affiliations:** 1 College of Animal Science, Xinjiang Agricultural University, Urumqi 830052, China; luobao0225@163.com (C.L.); wangdonghai0323@163.com (D.W.); yuxiong8763601@126.com (X.Y.); 2 College of Animal Science and Technology, China Agricultural University, Beijing 100193, China; luna19831227@163.com (N.L.); caozhijun@cau.edu.cn (Z.C.); yang_hongjian@sina.com (H.Y.); lisheng0677@163.com (S.L.); 3 Inner Mongolia Shengmu Animal Husbandry Co., Ltd., Hohhot 010018, China; lihaiqing@smorganic.cn (H.L.); liugaofei@smorganic.cn (G.L.)

**Keywords:** soybean cake, alfalfa hay, rumen degradation rate, amino acid

## Abstract

**Simple Summary:**

In this study, six organic feed raw materials (corn grain, soybean cake, wheat bran, corn silage, oat hay, and alfalfa hay) were selected for a comparison of their nutritional values. The chemical composition, amino acid content, and rumen degradation characteristics of these raw materials were studied in detail.

**Abstract:**

The current study was designed to investigate the chemical composition, amino acid content, and rumen degradation characteristics (nylon bag method) of six organic feeds to illustrate their feeding values. The feeds analyzed were: corn grain (CG), soybean cake (SC), wheat bran (WB), corn silage (CS), oat hay (OT), and alfalfa hay (AF). Our results showed that the contents of crude protein (CP) (47.46%) and ether extract (EE) (8.23%) in SC were highest. The contents of neutral detergent fiber (NDF) (65.00%) and acid detergent fiber (ADF) (39.16%) in OT were highest. The contents of total amino acid (TAA) (42.95%) and essential amino acid (EAA) (19.73%) in SC were highest. Among SC, WB, and CG, the effective degradation rate (ED) of dry matter (DM) is SC (87.89%) > WB (73.32%) > CG (64.25%); the ED value of CP is CG (82.37%) > WB (82.40%) > SC (60.47%). Among CS, OT, and AF, the effective degradation rate (ED) of DM is CS (72.68%) > OT (59.97%) > AF (58.89%); the ED value of CP is AF (76.46%) > CS (72.03%) > OT (71.99%). In conclusion, the chemical composition, amino acid content, and rumen degradation rate of SC and AF were better than those of the other four feeds.

## 1. Introduction

With changes in consumption levels, people’s choice of dairy products has become more and more diverse. Organic milk is more exposed to consumers because of its advantages, such as being safe, green, low emission, etc. [1]. It is estimated that by 2050, dairy consumption will increase by 58% compared with the current level [2]; therefore, the scale of organic milk production and organic farming will gradually expand.

Organic milk is produced under the organic breeding standard procedures, which pay particular attention to the welfare of dairy cows, relating to the overall requirements for breeding, environment, and processing [3]. For example, a certain proportion of organic raw material must be used in the feeding process, and the European Union even stipulates that organic feed should be used completely in the breeding of organic dairy cows [4,5]. Although this limits the use of many conventional feeds and additives to a certain extent [6], some data indicate that, compared with conventional production of organic herds, the feeding efficiency is higher [7], and the quality of livestock products produced under organic conditions is also higher [8]. Understanding the nutritional composition of organic feed used in organic production is necessary [9,10].

Crude protein (CP) is an important component of feed, and CP content and utilization were once an important basis for evaluating the quality of feed. However, the content of CP in the feed cannot be equal to the utilization rate of protein [11,12]. For example, the amino acid content and ratio of the feed will have an impact on the utilization of protein [13]. The utilization rate of protein is affected by many factors, and the final utilization rate needs to be judged based on actual digestive capacities. As a feed evaluation method, the nylon bag method can intuitively reflect the degradation effect of the feed in the body and is an important supplement to the utilization efficiency of CP content in feed [14,15]. Therefore, six kinds of organic feed commonly used in production were selected in this experiment and their chemical compositions, amino acid contents, and rumen degradation rates were detected to provide reference data for the rational utilization of organic feeds.

## 2. Materials and Methods

### 2.1. Raw Materials

Six types of organic feed (corn grain (CG), soybean cake (SC), wheat bran (WB), corn silage (CS), oat hay (OT), and alfalfa hay (AF)) from the same batch on six farms in one month were selected for this study. Each type of organic feed was provided by Inner Mongolia Shengmu Animal Husbandry Co., Ltd (Hohhot, China). All feeds were certified as organic. 

Six organic feeds were selected and every feed was sampled in triplicate. The crude protein (CP), crude ash (Ash), and dry matter (DM) content of the organic feeds were analyzed according to AOAC (1984) [16]. Neutral detergent fiber (NDF) and acid detergent fiber (ADF) were analyzed with a fiber analyzer (ANKOM 2000i, ANKOM Technology Co. Ltd., New York, NY, USA) according to the method of Van Soest et al. [17]. The amino acid content (AA) in organic feeds was analyzed with an AA analyzer (Hitachi High-Technologies Co. Ltd., Tokyo, Japan) according to the method of Winters et al. [18].

### 2.2. Animals and Experimental Design

Four Holstein cows (bodyweight: 592 ± 23 kg) with rumen fistulas were used for this test. The cows’ basic diet and its nutrient composition is presented in Table 1.

### 2.3. In Situ Nutrient Degradability

The nylon bag method [19] was used in this test to reanalyze the rumen degradation characteristics of DM and CP. Six kinds of organic feeds were put into nylon bags (size: 8 cm × 12 cm, pore: 50 μm) for incubation according to the operation adopted from Ma et al. [20]. According to the different incubation times, six kinds of samples were divided into two parts for testing, namely, concentrate and roughage. The incubation times of concentrate samples (CG, SC, WB) were 2 h, 4 h, 8 h, 16 h, 24 h, and 48 h; roughage samples (CS, OT, AF) were 4 h, 8 h, 12 h, 24 h, 30 h, 48 h, and 72 h. The nylon bags were washed with cold water till clear after incubation at each time, then dried (65 °C for 48 h) and milled (1 mm sieve) for analysis and calculation.

### 2.4. Calculation of In Situ Nutrient Degradability

The degradation data formula is as follows [21]:

(1) $$P=a+b\times \left(1-e^{-\mathrm{ct}}\right)$$

where P is the nutrient disappearance rate in the rumen at one time “t”; “a” is a rapidly degradable fraction; “b” is the potentially degradable fraction; “c” is the constant rate of degradation of “b”(%/h). The NLIN program in SAS (ver. 8.2; SAS Institute, Inc., Cary, NC, USA) was used to calculate a, b, and c. Each cow was regarded as a duplicate.

Then, the effective degradability (ED) formula is as follows [21]:

(2) ED(%)=a+b×c/(k+c)

where “k” is the rumen outflow rate of the nutrient component (%/h). In this study, the value of k was 0.00139 + 0.17758 × c (%/h) [22].

### 2.5. Statistical Analysis

Data were expressed as the means and standard deviations. Amino acid content, rumen real-time degradability, and degradation parameters were subjected to SAS with the following model (3):

(3) Yijk=μ+Fi+Tj+(F×T)ij+Aik+εijk 

where “Fi” is the fixed effect of feeds, “Tj” is the fixed effect of incubation time, and “(F × T)ij” is the fixed interaction effect between organic feeds and incubation time. “Ak” is the random effect of the animals, and “εijk” is the error.

For all statistical analyses in this study, significance was declared at *p* < 0.05. Differences were evaluated using a multiple comparison test following the Tukey–Kramer method.

## 3. Results

### 3.1. Chemical Composition of Organic Feed 

The chemical composition of six organic feed samples is presented in Table 2. Among the six organic feeds, SC has the highest content of CP and EE, AF has the highest ash content, and OT has the highest NDF and ADF content. Among the three organic concentrates, the content of CP, EE, and Ash in SC is higher than that of WB and CG, while the content of NDF and ADF in WB is higher than that of SC and CG. In the three organic roughages, the content of CP, EE, and Ash of AF is higher than that of OT and CS, while the content of NDF and ADF of OT is higher than that of AF and CS.

### 3.2. Amino Acid Composition

The amino acid composition of six organic feed samples is presented in Table 3. Among the six feeds, the contents of EAA, NEAA, and TAA in SC were significantly (*p* < 0.05) higher than those of the other raw materials, while the opposite was the case for CS. Among the three types of concentrates, the contents of EAA, NEAA, and TAA in CG were significantly (*p* < 0.05) lower than those in WB and SC; among the three types of roughage, the contents of EAA, NEAA, and TAA of AF were significantly (*p* < 0.05) higher than those of OT and CS.

### 3.3. Ruminal DM Degradation

**Figure 1:**
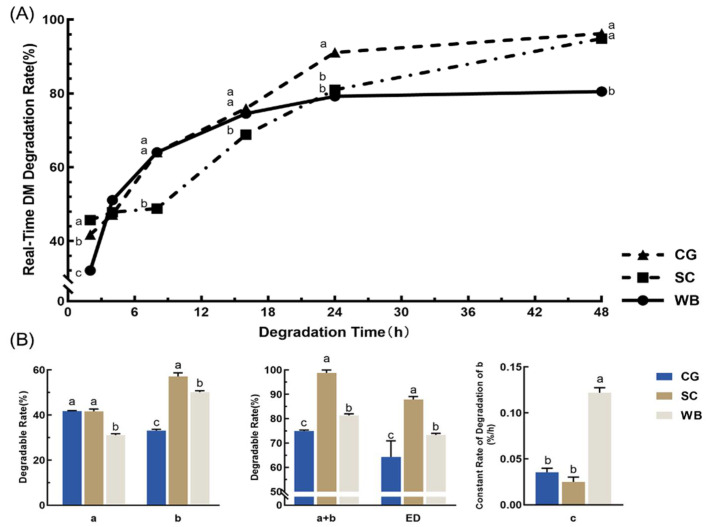
Rumen real-time degradability and degradation parameters of DM for three organic concentrates (CG, SC, and WB). (**A**) Real-time degradation rates. (**B**) Degradation parameters. Data in the same parameter (in (**B**)) or time points (in (**A**)) of the superscript mark different lowercase letters indicate a significant difference (*p* < 0.05). “a” (%): rapidly degradable fraction; “b” (%): the potentially degradable fraction; “c” (%/h): the constant rate of degradation of “b” (%/h); ED (%): effective degradability.

The rumen real-time degradability and degradation parameters of DM for three organic concentrates are presented in Figure 1. At 24 h, the degradation of CG was 91.06%, which is significantly (*p* < 0.05) higher than the degradations of SC and WB. The 48 h degradations of CG and SC were the highest—96.20% and 94.86%, respectively. The rapidly degradable fraction (a) of CG and SC was significantly higher than WB (*p* < 0.05). The potentially degradable fraction (b) of SC was significantly (*p* < 0.05) higher than CG and WB. The effective degradation (ED) of SC was significantly higher than CG and WB.

**Figure 2:**
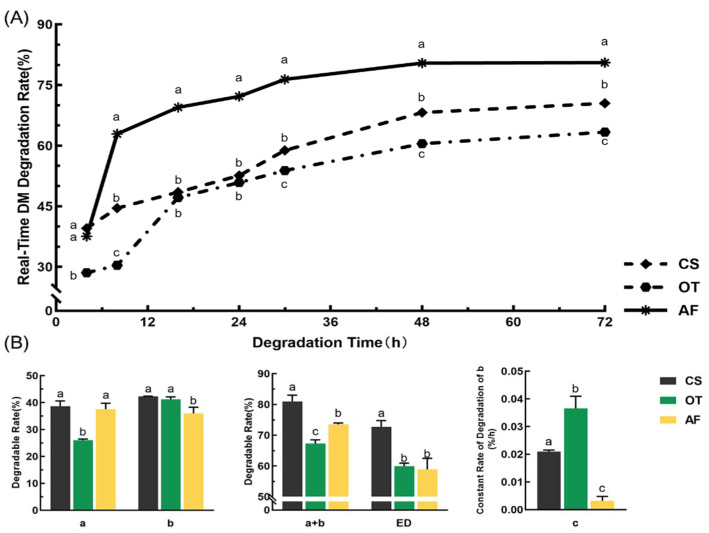
Rumen real-time degradability and degradation parameters of DM for three organic roughages (CS, OT, and AF). (**A**) Real-time degradation rates. (**B**) Degradation parameters. Data in the same parameter (in (**B**)) or time points (in (**A**)) of the superscript mark different lowercase letters indicate a significant difference (*p* < 0.05). “a” (%): rapidly degradable fraction; “b” (%): the potentially degradable fraction; “c” (%/h): the constant rate of degradation of “b” (%/h); ED (%): effective degradability.

The rumen real-time degradability and degradation parameters of DM for three organic roughages are presented in Figure 2. At 8 h, 16 h, 24 h, 30 h, 48 h, and 72 h, the rumen degradations of AF were significantly (*p* <0.05) higher than those of OT and CS. At 30 h and 72 h, the degradations of OT were significantly lower (*p* < 0.05) than those of AF and CS. 

The rapidly degradable fraction (a) of CS and AF was significantly higher (*p* < 0.05) than OT. The potentially degradable fraction (b) of CS and OT was significantly higher than AF (*p* < 0.05). The effective degradation (ED) of CS was 72.68%, which was significantly (*p* < 0.05) higher than AF and OT.

### 3.4. Ruminal CP Degradation

**Figure 3:**
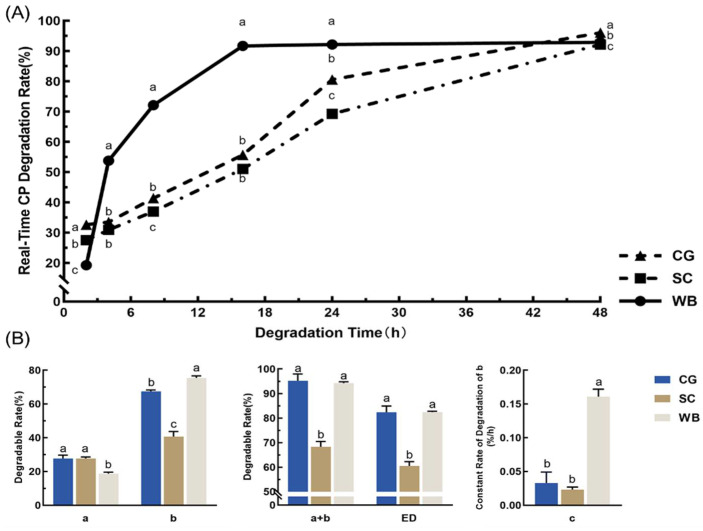
Rumen real-time degradability and degradation parameters of CP for three organic concentrates (CG, SC, and WB). (**A**) Real-time degradation rates. (**B**) Degradation parameters. Data in the same parameter (in (**B**)) or time points (in (**A**)) of the superscript mark different lowercase letters indicate a significant difference (*p* < 0.05). “a” (%): rapidly degradable fraction; “b” (%): the potentially degradable fraction; “c” (%/h): the constant rate of degradation of “b” (%/h); ED (%): effective degradability.

The rumen real-time degradability and degradation parameters of CP for three organic concentrates are presented in Figure 3. At 4 h, the CP degradations of CG and SC were similar at 30–34%, which was significantly (*p* < 0.05) lower than WB. At 16 h, the CP degradation of WB was 91.71%, which was significantly (*p* < 0.05) higher than those of CG and SC. At 24 h, the degradation of WB was still significantly (*p* < 0.05) higher than those of CG and SC. At 48 h, the degradation of the three organic concentrates all reached more than 90%. Among them, the degradation of CG was significantly (*p* < 0.05) higher than that of SC and WB, being 96.03%

The rapidly degradable fraction (a) of CG and SC was significantly higher than WB (*p* < 0.05). The potentially degradable fraction (b) of WB was significantly (*p* < 0.05) higher than CG and SC. The effective degradation (ED) of CG and WB was around 82%. The effective degradation (ED) of SC was significantly (*p* < 0.05) lower than CG and WB.

**Figure 4:**
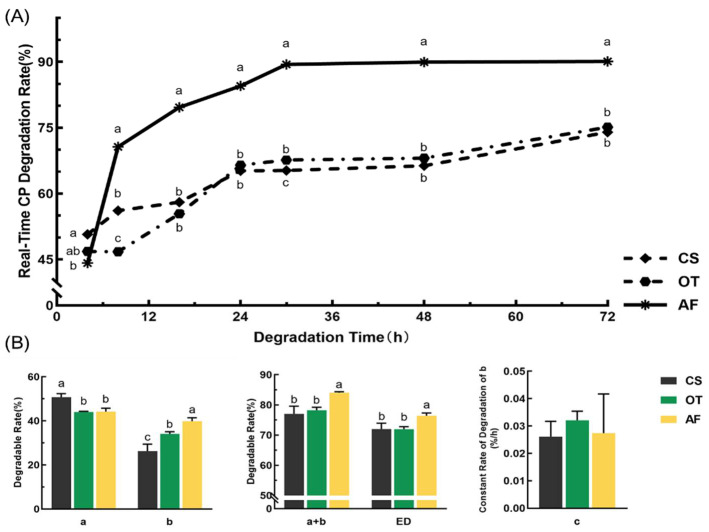
Rumen real-time degradability and degradation parameters of CP for three organic roughages (CS, OT, and AF). (**A**) Real-time degradation rates. (**B**) Degradation parameters. Data in the same parameter (in (**B**)) or time points (in (**A**)) of the superscript mark different lowercase letters indicate a significant difference (*p* < 0.05). “a” (%): rapidly degradable fraction; “b” (%): the potentially degradable fraction; “c” (%/h): the constant rate of degradation of “b” (%/h); ED (%): effective degradability.

The rumen real-time degradability and degradation parameters of CP for three organic roughages are presented in Figure 4. At 4 h, the CP degradation of CS was significantly (*p* < 0.05) higher than AF. However, the degradation of AF was significantly (*p* < 0.05) higher than OT and CS at 8 h, 16 h, 24 h, 30 h, and 48 h. The degradations of three roughages stabilized at 30 h. At 72 h, the degradations of AF were significantly (*p* < 0.05) higher than CS and OT, but the degradations between CS and OT were not significantly different (*p* > 0.05).

The rapidly degradable fraction (a) of CS was significantly (*p* < 0.05) higher than OT and AF. The potentially degradable fraction (b) of AF was significantly (*p* < 0.05) higher than OT and CS; the potentially degradable fraction (b) of CS was significantly *(p* < 0.05) lower than OT and AF. The effective degradation (ED) of AF was significantly (*p* < 0.05) higher than OT and CS, but there was no significant difference (*p* > 0.05) between OT and CS.

## 4. Discussion

Alfalfa hay (AF) is a high-quality pasture due to the content of high-quality protein and fiber [23,24]. In this study, the CP content of AF was 19.61%, which was consistent with the second-class alfalfa hay described by Inal et al. [25], indicating that organic AF was equivalent to conventional alfalfa hay in terms of CP. The content and ratio of amino acids also have an important impact on the nutritional value of protein [26]. Amino acid content is an important indicator for judging the protein nutrition of roughage. Protein content and composition can directly affect the growth of the cattle [27] and can also indirectly affect nitrogen metabolism [28]. Our study showed that the EAA content of SC is the highest, or it can be used as a source of high-quality EAA to adjust the balance of amino acids in the diet. The ratio between EAA and NEAA can be used to describe the quality of roughage, especially when the ratio is greater than 60% [29]. The study showed the ratios between EAA and NEAA for three roughages (CS, OT, and AF) as being greater than 60%, which reveals that they can be used as high-quality protein and amino acid sources for dairy cows. 

As an approach to evaluating the difficulty of feed utilization, the in situ nylon bag method is often used to study the degradation and utilization efficiency of feed in the rumen. The DM degradations reflect the digestion of DM in the feed by the rumen within a certain period, reflecting the overall degradation of the feed in the rumen and the amount of nutrients the rumen could take in the feed. This study showed that the 48 h DM degradations of CG and SC were both higher than 90%, which reveals that the rumen utilization rate of CG and SC is high. The 48 h DM degradation of CG is higher than those reported in the studies by Li et al. (88.04%) [30] and Zhu et al. (76.08%) [31]. The study showed that the 48 h DM degradation of WB reached 80.48%, which is higher than those in the studies by Dong et al. (73.21%) [32] and Das et al. (73.33%) [33]. The DM degradation values of three organic concentrates (CG, SC, and WB) reveal that the overall rumen degradation of organic concentrates is better than that of conventional concentrates. Starch degradation is positively correlated with DM degradation [34], possibly because the starch or soluble protein contents of the organic feed DM are higher than those of conventional feed, though this may also be caused by different varieties.

In this study, the DM degradation of AF and CS at 72 h was similar to the report by Liu et al. [35]. This reveals that the rumen DM degradation of AF and CS is the same as conventional AF and SC. In this study, the 72 h DM degradation of OT was lower than in the study by Turgut et al. (70.8%) [36] but higher than in the study by Liu et al. (63.17%) [37]. This reveals that the rumen DM degradations of organic OT were in a reasonable range. 

The content of CP in feed cannot be equated with the actual protein utilization of the animal. For example, fiber level [11] and anti-factor factors [38] will affect the utilization efficiency of CP. By measuring the degradation rate of CP in the rumen of the feed, the true protein digestion level of the feed can be understood and the feed can be used rationally, thus waste can be avoided. This study showed that the CP degradations at 48 h of the three organic concentrates (CG, SC, and WB) were all higher than 92%, which reveals that the organic concentrates’ CP contents can be better utilized by the rumen. The CP degradation of CG at 48 was higher than in the studies by Son et al. (82.6%) [39] and Zhu et al. (76.08%) [31]. The CP degradations of WB at 48 h have no significant difference from the values determined by Karimi et al. [40]. This study showed that the CP degradation of organic CG is much higher than conventional corn grain, while WB is not significantly different from conventional feed. In this study, the 48 h CP degradations in SC were similar to those in the study by Moghadam et al. [41], which indicates that there is no significant difference between SC and conventional soybean cake. However, the CP degradation of SC at 24 h was significantly lower than the CP degradation rate of CG and WB in the same time period. This may be related to the characteristics of SC, which can be used to develop ruminal bypass protein production.

This study shows that the CP degradation of AF in 72 h was 90.10%, and it had already reached 89.40% at 30 h, which was higher than the range of previous studies (80–86%) [35,37,42]. This result indicates that AF had a better degradation and could be degraded quickly. The utilization efficiency of CP is relatively high. The molecular weight and stability of the soluble protein in AF will change CP degradation in the rumen [43]. In this study, the amino acid content of AF was different from that of conventional alfalfa hay [44]; changes in amino acid content may cause changes in soluble protein, leading to an increase in CP degradation rate. In this study, the 72 h CP degradation of OT was not significantly different from that of CS, which shows that OT is similar to CS in terms of protein utilization.

## 5. Conclusions

In conclusion, SC had the highest CP and OT had the highest Ash, NDF, and ADF contents. Among three concentrates (SC, CG, and WB), the effective degradability (ED) of DM was highest in SC; the ED of CP was highest in WB. Among three roughages (AF, OT, and CS), the ED of DM was highest in CS; the ED of CP was highest in AF.

## Figures and Tables

**Table 1:** Composition and nutrient value of basic diet (DM basis %).

Ingredients	Content	Nutrient Levels ^2^	Content
Whole corn silage	30.76	NE_L_(MJ/kg)	1.78
Alfalfa hay	11.88	CP	16.98
Corn	11.49	NDF	65.26
Steam-flaked corn	15.72	ADF	14.73
Soybean meal	13.74	EE	3.77
Soybean hull	3.89	Ash	8.81
Cottonseed	1.90	Ca	0.86
Molasses	3.33	P	0.38
Corn gluten meal	2.72		
Rumen-protected fatty acid	1.47		
Premix ^1^	2.51		
NaHCO_3_	0.60		

^1^ Each kilogram of premix contains 1,000,000 IU/kg vitamin A, 3,280,000 IU/kg vitamin D, 10,000 IU/kg vitamin E, 1000 mg/kg nicotinic acid, 0.6 mg/kg copper, 1.2 mg/kg zinc, 2.2 mg/kg manganese, 76 mg/kg iodine, 5.5 mg/kg selenium, and 29 mg/kg cobalt. ^2^ NE_L_ is a calculated value, while the other nutrient levels were test values.

**Table 2:** Chemical composition of six kinds of organic feed (% DM basis).

Items	DM	CP	EE	Ash	NDF	ADF
SC	89.99 ± 0.08 ^c^	47.46 ± 0.07 ^a^	8.23 ± 0.06 ^a^	6.77 ± 0.03 ^c^	17.56 ± 0.65 ^c^	8.06 ± 0.04 ^e^
WB	86.43 ± 0.03 ^d^	20.15 ± 0.06 ^b^	2.97 ± 0.05 ^b^	5.67 ± 0.01 ^d^	43.72 ± 1.15 ^b^	12.82 ± 0.16 ^d^
CG	85.96 ± 0.12 ^e^	8.65 ± 0.02 ^e^	2.23 ± 0.01 ^c^	1.37 ± 0.04 ^f^	8.07 ± 0.35 ^d^	2.11 ± 0.43 ^f^
OT	95.04 ± 0.00 ^a^	8.88 ± 0.13 ^d^	0.74 ± 0.10 ^e^	8.55 ± 0.27 ^b^	65.00 ± 1.99 ^a^	39.16 ± 0.43 ^a^
AF	93.86 ± 0.06 ^b^	19.61 ± 0.02 ^c^	1.38 ± 0.08 ^d^	11.50 ± 0.04 ^a^	44.84 ± 1.63 ^b^	31.47 ± 0.42 ^b^
CS	31.30 ± 0.14 ^f^	7.99 ± 0.06 ^f^	2.25 ± 0.06 ^c^	5.48 ± 0.07 ^e^	42.91 ± 0.13 ^b^	23.63 ± 0.09 ^c^

CG: Corn grain, SC: Soybean cake, WB: Wheat bran, CS: Corn silage, OT: Oat hay, AF: Alfalfa hay, DM: Dry matter, CP: Crude protein, NDF: Neutral detergent fiber, ADF: Acid detergent fiber. Data in the same column of the superscript mark different lowercase letters indicate a significant difference (*p* < 0.05).

**Table 3:** Amino acid composition of six kinds of organic feed (%DM basis).

Amino Acid	Samples					
	CG	SC	WB	CS	OT	AF
Arg	0.36 ± 0.01 ^d^	3.37 ± 0.07 ^a^	1.12 ± 0.01 ^b^	0.18 ± 0.00 ^e^	0.28 ± 0.01 ^d^	0.73 ± 0.02 ^c^
His	0.20 ± 0.01 ^d^	1.21 ± 0.03 ^a^	0.44 ± 0.01 ^b^	0.13 ± 0.00 ^e^	0.11 ± 0.00 ^e^	0.36 ± 0.00 ^c^
Ile	0.26 ± 0.01 ^d^	1.99 ± 0.04 ^a^	0.50 ± 0.02 ^c^	0.27 ± 0.00 ^d^	0.29 ± 0.00 ^d^	0.72 ± 0.01 ^b^
Leu	0.91 ± 0.02 ^d^	3.47 ± 0.06 ^a^	1.02 ± 0.00 ^c^	0.77 ± 0.01 ^e^	0.54 ± 0.00 ^f^	1.23 ± 0.03 ^b^
Lys	0.23 ± 0.01 ^e^	2.77 ± 0.06 ^a^	0.70 ± 0.00 ^c^	0.25 ± 0.01 ^e^	0.35 ± 0.00 ^d^	1.11 ± 0.00 ^b^
Met	0.15 ± 0.00 ^c^	0.46 ± 0.00 ^a^	0.23 ± 0.00 ^b^	0.11 ± 0.00 ^d^	0.09 ± 0.00 ^e^	0.23 ± 0.00 ^b^
Phe	0.43 ± 0.01 ^d^	2.09 ± 0.02 ^a^	0.60 ± 0.01 ^c^	0.35 ± 0.00 ^e^	0.43 ± 0.01 ^d^	0.79 ± 0.03 ^b^
Thr	0.27 ± 0.01 ^d^	1.76 ± 0.04 ^a^	0.54 ± 0.00 ^c^	0.29 ± 0.01 ^d^	0.30 ± 0.00 ^d^	0.77 ± 0.01 ^b^
Trp	0.05 ± 0.00 ^e^	0.53 ± 0.02 ^a^	0.26 ± 0.00 ^b^	0.05 ± 0.00 ^e^	0.08 ± 0.00 ^d^	0.24 ± 0.01 ^c^
Val	0.34 ± 0.01 ^d^	2.09 ± 0.04 ^a^	0.77 ± 0.00 ^c^	0.37 ± 0.00 ^d^	0.37 ± 0.00 ^d^	0.91 ± 0.00 ^b^
Lys/Met	1.53 ± 0.03 ^f^	6.07 ± 0.08 ^a^	2.99 ± 0.04 ^d^	2.31 ± 0.00 ^e^	3.79 ± 0.04 ^c^	4.74 ± 0.05 ^b^
EAA	3.20 ± 0.07 ^de^	19.73 ± 0.35 ^a^	6.18 ± 0.00 ^c^	2.77 ± 0.02 ^ef^	2.85 ± 0.03 ^de^	7.90 ± 0.02 ^b^
Ala	0.54 ± 0.02 ^d^	2.00 ± 0.04 ^a^	0.79 ± 0.00 ^c^	0.77 ± 0.01 ^c^	0.40 ± 0.00 ^e^	0.94 ± 0.00 ^b^
Asp	0.52 ± 0.02 ^e^	5.04 ± 0.11 ^a^	1.17 ± 0.02 ^c^	0.39 ± 0.02 ^f^	0.68 ± 0.00 ^d^	2.15 ± 0.00 ^b^
Cys	0.14 ± 0.00 ^d^	0.58 ± 0.01 ^a^	0.33 ± 0.01 ^b^	0.10 ± 0.00 ^e^	0.10 ± 0.00 ^e^	0.22 ± 0.00 ^c^
Glu	1.35 ± 0.03 ^d^	7.90 ± 0.17 ^a^	3.12 ± 0.05 ^b^	0.82 ± 0.01 ^e^	0.87 ± 0.00 ^e^	1.65 ± 0.01 ^c^
Gly	0.27 ± 0.00 ^d^	1.96 ± 0.04 ^a^	0.88 ± 0.01 ^b^	0.34 ± 0.00 ^c^	0.32 ± 0.00 ^c^	0.79 ± 0.00 ^b^
Pro	0.69 ± 0.01 ^d^	2.04 ± 0.06 ^a^	1.07 ± 0.02 ^c^	0.52 ± 0.01 ^e^	0.53 ± 0.02 ^e^	1.19 ± 0.03 ^b^
Ser	0.36 ± 0.01 ^d^	2.30 ± 0.05 ^a^	0.72 ± 0.01 ^c^	0.24 ± 0.01 ^e^	0.31 ± 0.00 ^d^	0.81 ± 0.01 ^b^
Tyr	0.31 ± 0.01 ^d^	1.39 ± 0.02 ^a^	0.44 ± 0.00 ^c^	0.20 ± 0.01 ^f^	0.25 ± 0.01 ^e^	0.48 ± 0.01 ^b^
NEAA	4.17 ± 0.08 ^c^	23.22 ± 0.50 ^a^	8.52 ± 0.13 ^b^	3.38 ± 0.00 ^d^	3.48 ± 0.02 ^d^	8.22 ± 0.01 ^b^
TAA	7.37 ± 0.15 ^c^	42.95 ± 0.85 ^a^	14.70 ± 0.12 ^b^	6.14 ± 0.00 ^d^	6.64 ± 0.01 ^d^	15.31 ± 0.01 ^b^

EAA: Essential amino acid, NEAA: Non-essential amino acid, TAA: Total amino acids, Arg: Arginine, His: Histidine, Ile: Isoleucine, Leu: Leucine, Lys: Lysine, Met: Methionine, Ala: Alanine, Thr: Threonine, Trp: Tryptophan, Val: Valine, Asp: Aspartate, Cys: Cystine, Glu: Glutamic acid, Pro: Proline, Ser: Serine, Tyr: Tyrosine. Data in the same column of the superscript mark different lowercase letters indicate a significant difference (*p* < 0.05).

## Data Availability

All the data are already provided in the main manuscript.
